# Research Progress on Fiber-Reinforced Recycled Brick Aggregate Concrete: A Review

**DOI:** 10.3390/polym15102316

**Published:** 2023-05-16

**Authors:** Zhenya Zhang, Yongcheng Ji, Dayang Wang

**Affiliations:** School of Civil Engineering and Transportation, Northeast Forestry University, Harbin 150040, China

**Keywords:** recycled brick aggregate concrete, mechanical properties, glass fiber, polypropylene fiber

## Abstract

The addition of fibers to strengthen recycled concrete can strengthen the inherent deficits and deficiencies of concrete containing recycled aggregates to some extent and enlarge the concrete’s application range. In order to further promote the development and application of fiber-reinforced brick aggregate recycled concrete, the research results regarding its mechanical properties are reviewed in this paper. The effect of the content of broken brick on the mechanical properties of recycled concrete and the effects of different categories and contents of fiber on the basic mechanical properties of recycled concrete are analyzed. The problems to be solved in research on the mechanical properties of fiber-reinforced recycled brick aggregate concrete are presented, and the related research suggestions and prospects are summarized. This review provides a reference for further research in this field and the popularization and application of fiber-reinforced recycled concrete.

## 1. Introduction

With the rapid development of the economy, a large amount of construction waste is produced in the processes of demolition and reconstruction of old buildings and the construction of new buildings, causing severe damage to the ecological environment and produced a large amount of waste. In a 2019 assessment alone, construction waste in the United States and China was found to be approximately 548 million tons and 250 billion tons, respectively [1]. Concrete (60 percent), brick (21 percent), and mortar (9 percent) constitute the most significant proportion of significant construction waste [2]. Recycled concrete aggregate (RCA) is a mixture of waste concrete in complex conditions after crushing, cleaning, screening, and proportioning. As one of the primary construction wastes, if it can be used to produce recycled concrete, it will have great environmental significance: while effectively recycling building resources, it will reduce the damage to and pollution of the natural environment, which is in line with the national environmental policy.

There are many types of research on the mechanical properties of brick aggregate recycled concrete, which mainly focus on physical and mechanical properties, such as compressive properties, flexural properties, elastic modulus, and splitting tensile properties. Most experiments are carried out to explore changes in different brick aggregate or brick concrete aggregate contents. The mechanical properties of recycled concrete tend to decline with the increase in the replacement rate of brick aggregate, including the compressive strength, flexural strength, elastic modulus, and splitting tensile strength [3,4,5,6,7,8,9,10,11,12]. Among this research, there are many in-depth studies. For example, Liu Lanjun [5] et al. studied the relationship between the 28-day compressive strength, 28-day relative bending strength, 28-day relative splitting tensile strength, and the content of broken bricks based on the results of a regression analysis. Furthermore, Hashempour et al. [10] proposed the stress–strain relationship of recycled concrete containing red brick. However, according to this research, when the red brick content does not exceed 10~25%, the compressive strength reduction will not exceed 4.86%, having little influence on the mechanical properties of recycled concrete [13,14].

However, some defects in brick aggregate recycled concrete restrict its application in engineering. For example, due to the high porosity of brick aggregate, more water needs to be added during mixing to ensure the concrete’s workability. As a result, the water absorption rate of reclaimed aggregate is much higher than that of natural aggregate, which means that the shrinkage deformation of reclaimed concrete is more significant when water is lost, and the durability of reclaimed concrete is inferior to that of natural aggregate concrete. The experimental results show that the water absorption of recycled aggregate is approximately 15 times that of natural aggregate [15]. For instance, the permeability of recycled concrete is excellent [16]. However, because the fastened mortar is fragile and porous, the resistance of recycled concrete to sulfate and acid corrosion could be better, with poor abrasion resistance, low compressive strength, low density, high water absorption, and other defects [17]. In order to overcome the performance defects of recycled aggregate, many researchers have attempted to blend different fibers. For example, combining polyvinyl alcohol fiber and MgO can effectively solve the problem of concrete cracking and abrasive damage [18]. Akeed et al. [19] studied the impermeability of fiber-reinforced concrete and the effect of fiber-reinforced concrete on chloride ion permeability. Many researchers have also studied waste fiber recycled concrete’s splitting tensile strength and flexural properties under the influence of different recycled aggregate contents, fiber lengths, and water–cement ratios. The results show that adding fiber improves the strength and toughness of recycled concrete [20,21,22].

Glass fiber is cheap and lightweight and has good tensile strength, and it has become a focal material in fiber-modified reclaimed concrete research. As early as the 1950s, the former Soviet Union began to use glass fiber as a modified material in concrete [23]. However, the glass fiber was not corrosion-resistant at this time, resulting in durability problems. Nevertheless, with the development and improvement of alkali-resistant glass fiber and its exploratory application in structures, glass-fiber-reinforced concrete can be used as a load-bearing member [24,25,26].

Most current research focuses on the basic mechanical properties of glass-fiber-reinforced recycled concrete at room temperature. Ali et al. [27] studied the effect of glass fiber (GF) on recycled coarse aggregate concrete’s mechanical properties and durability. The test results showed that the recycled concrete containing 0.25% glass fiber and 50% recycled aggregate had a better splitting tensile strength and bending strength than ordinary concrete. However, the addition of glass fiber negatively affected the anti-seepage property. Many researchers have investigated the effect of glass fiber on the performance of recycled concrete in depth. It was found that a concrete-filled glass-fiber-reinforced polymer pipe had a good ductility and energy dissipation capacity under repeated loading [28]. At the same time, the addition of fly ash, fiber, and MgO could also improve the frost resistance of concrete [29].

Based on previous research, many more in-depth studies have been conducted on the mechanism of fiber-modified concrete. Wang et al. studied the effects of polypropylene (PP), polyvinyl alcohol (PVA), and polyacrylonitrile (PAN) fibers of varying lengths (10 mm and 20 mm) on the workability, strength, shrinkage, cracking resistance, and durability of panel concrete [30]. For example, the addition of glass fiber (GF) or polypropylene fiber (PFF) can bridge microcracks in the matrix, redistribute tension, and prevent stress diffusion at the crack tip [31,32,33,34] and has an excellent inhibitory effect on the matrix in the crack initiation and crack propagation stages. Xu [35] and other researchers found that fiber-modified concrete can increase the corresponding load-bearing capacity after the peak load to attain the “Cracking but not breaking” failure mode [36,37,38], thus effectively improving the mechanical properties of concrete, including its tensile strength, bending strength, toughness, ductility, modulus of fracture, and energy absorption [39,40,41,42,43,44,45,46,47,48,49,50,51,52,53], described in a long list of properties. Enhancing the mechanical properties of concrete using GF and PPF from brittle material through extension dramatically improves the toughness of the concrete matrix [48,49,54], expanding the application scope of concrete structures. Some studies have revealed that GF or PFF can effectively regulate plastic shrinkage cracking [55,56] and drying shrinkage strain [42,44,57] and improve freeze–thaw resistance [58,59,60]. This research is of great practical significance to the north of China, which has long and cold winters, and can significantly extend the service life of concrete structures.

Research on polypropylene fiber-modified brick aggregate recycled concrete is also expanding. Several studies have found that [61,62,63] the addition of polypropylene fiber causes the microstructure of recycled concrete to undergo densification. To a certain extent, the addition of a certain amount of polypropylene fiber can enhance the compressive, tensile, shear, and other mechanical properties of recycled concrete brick aggregate [64,65]. Zhang [66] and other researchers conducted a comparative study on the mechanical properties and durability of fiber-reinforced concrete formed of a coarse aggregate of broken brick and natural aggregate and found that fiber could not only enhance the mechanical properties of concrete but also enhance the mechanical properties, and it could also improve the water resistance. Several studies have demonstrated that the addition of PPF decreases air or water permeability [67,68,69] and water absorption [70,71,72,73,74] and increases the impermeability of chloride ions [74]. PPF can reduce the permeability and capillary porosity through the pore-filling effect of the action mechanism, substantially improve the microstructure of concrete, and consequently improve the durability of the concrete structure [75,76].

In summary, the formation of recycled concrete with waste broken brick as a coarse aggregate is of immense environmental significance. Instead, there is great variation in how the addition of waste broken brick as a coarse aggregate can affect the mechanical properties, making its practical use difficult. This paper mainly introduces the state of research on the mechanical properties of fiber-reinforced brick aggregate recycled concrete in China and other countries. This paper analyzes the present state of research on recycled concrete from the perspectives of the content of waste broken brick and the influences of the type and content of fiber on the mechanical properties of recycled concrete based on a summarization of the effects of polypropylene fiber and glass fiber on RAC. The possibilities and prospects for the development of fiber-modified brick aggregate recycled concrete are discussed.

## 2. Materials

**Figure 1:**
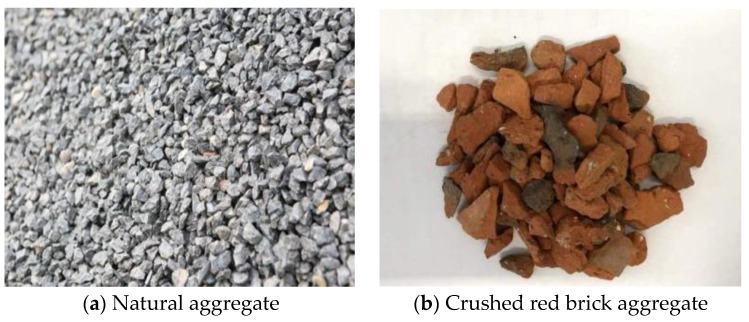
Coarse aggregate.

Aggregate with a particle size larger than 4.75 mm [77] is called coarse aggregate, divided into natural aggregate (NA) and recycled aggregate (RA). This paper predominantly discusses the effect of crushed brick as a recycled coarse aggregate on the performance of concrete. Two kinds of natural aggregate are used in daily life: crushed stone and cobblestone. The crushed stone is shown in Figure 1a. Crushed stone is natural rock or rock particles with a size above 4.75 mm which is produced through mechanical crushing and screening. Pebbles are rock particles larger than 4.75 mm in diameter, formed through natural weathering, water transportation, sorting, and stacking. Figure 1b shows that the crushed brick aggregate is a clay brick with particles larger than 4.75 mm after the crushing, cleansing, and screening of the waste brick from the brick/concrete construction site.

### 2.1. Coarse Aggregate of Clay Brick

After crushing, cleaning, and sieving, the waste bricks from the demolition site are mixed with gradation in proportions to form an aggregate called recycled brick aggregate. Concerning the coarse concrete aggregate, some attention must be paid to performance factors in the engineering application, including the volume density, apparent density, water absorption, and crushing value.

As shown in Table 1, compared with natural coarse aggregate, broken red brick aggregate (BBA) possesses a lower apparent density and bulk density, with a 19~40% lower apparent density and 20~47% lower bulk density, and a higher water absorption and crushing index, the water absorption being 11 to 49 times higher. The crushing index is 2.2 to 4 times higher, indirectly indicating that the strength and stability of red brick coarse aggregate are worse than those of natural aggregate. Compared with natural aggregate concrete (NAC), recycled brick aggregate concrete (RAC) coarse aggregate has a high porosity and crushing index. The higher porosity of BBA is the primary reason for its lower density. The surface of BBA is very rugged and possesses many pits and pores with diameters ranging from several microns to tens of microns. The loose structure formed of numerous pores and micro-cracks is the primary reason for the high crushing index of BBA. The crushing index is an important index used to measure coarse aggregate performance. It can be seen from the data in the table that the crushing value of a crushed brick aggregate is approximately four times that of natural aggregate. This indicates that the crushed brick aggregate has lower strength and higher porosity. Therefore, RAC made from BBA will have a lower compression capacity than concrete made from NA, which is a significant problem to overcome for brick aggregate recycled concrete.

### 2.2. Fiber Type

**Figure 2:**
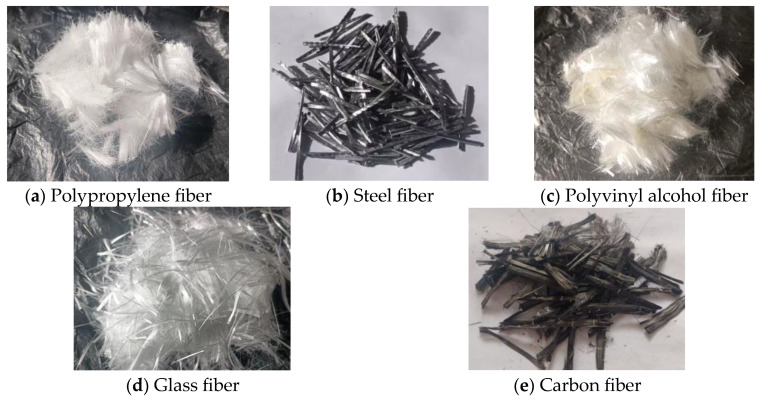
Common fibers.

The mechanical properties of BBA (such as the compressive strength, shear strength, and toughness) are typically lower than those of NA, making it challenging to popularize BBA in engineering applications. However, the addition of fiber can enhance the strength of RAC, mainly its crack resistance. At present, steel fiber (SF), polypropylene fiber (PF), carbon fiber (CF), polyvinyl alcohol fiber (PVA), and glass fiber (GF) are the main fiber types in this research field (Figure 2). The physical and mechanical properties of these five types of fibers are shown in Table 2. This paper mainly discusses the modification effects of glass and polypropylene fibers on RAC. Polypropylene fiber (PPF) is a synthetic fiber made of isotactic polypropylene, which is lightweight and has a low stiffness, corrosion resistance, high toughness, and low cost. It is a fiber with a wide range of uses and has become a focal point in the research and application of concrete reinforcement and toughening [82,83].

Glass fiber is made from waste glass or glass balls via high-temperature melting, drawing, winding, weaving, and other processes. Its diameter ranges from a few microns to 20 microns, equal to 1/20 to 1/5 the diameter of human hair. Each bundle of filaments consists of hundreds or even thousands of filaments. As a result, the utility model possesses the advantages of being lightweight and having good insulation, strong heat resistance, good corrosion resistance, high tensile strength, and a high elastic modulus [84].

**Table 2:** Mechanical properties of various fibers [61,85,86,87,88,89].

Fiber Type	Length (mm)	Aspect Ratio	Density (g/cm^3^)	Tensile Strength (MPa)	Young’s Modulus (GPa)
Polypropylene fiber (PPF)	14	400	0.91~0.92	276~700	3.5~9
Steel fiber (SF)	35	60	7.8~7.85	≥1150	200
Polyvinyl alcohol fiber (PVA)	35	450	0.91~1.29	1500~1900	40
Glass fiber (GF)	15	1500	0.91~2.36	350~1500	70~95
Carbon fiber (CF)	15	214	1.76	≥3000	205

Table 2 shows that the elastic modulus of polypropylene fiber is lower than that of other fibers. It belongs to the category of synthetic fiber with a low elastic modulus and has high construction performance. However, it is also widely used in marked concrete areas such as roofs and ground, usually in places that will not crack [88]. On the other hand, polypropylene fiber’s density is less than one-eighth that of steel fiber. Its tensile strength can reach 0.6 times that of the latter. The elastic modulus of glass fiber is greater than that of PPF and PVA, and the density is less than one-third that of steel fiber, but the tensile strength can even exceed that of steel fiber. It can be observed that polypropylene fiber and glass fiber are both excellent modified fibers.

## 3. Study on the Mechanical Properties of Recycled Concrete Containing Crushed Brick Aggregate

The mechanical properties of recycled concrete are an essential index reflecting the quality of recycled concrete. For instance, using broken red brick as a coarse aggregate significantly impacts the strength of recycled concrete, and it is essential to study its change law.

### 3.1. Compressive Strength

**Figure 3:**
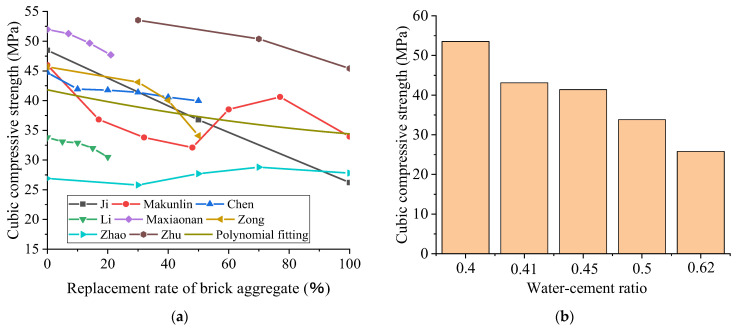
The effect of the replacement ratio of BBA on the compressive strength of recycled concrete (**a**) and the effect of the water–cement ratio on the cubic compressive strength of recycled concrete when the replacement ratio of BBA is 30% (**b**) [3,4,6,7,8,9,80,90].

The influence of the replacement rate of recycled brick aggregate on the compressive strength of RBC has yet to be fully determined by domestic and foreign researchers. It can be seen from Figure 3a that, in general, with the increase in the replacement rate of brick aggregate, the effect of the replacement rate of recycled brick aggregate on the compressive strength of RBC is significant, and the compressive strength of recycled concrete tends to decrease because of the development of surface defects in the recycled coarse aggregate. Moreover, the regenerated coarse aggregate surface shows old mortar adhesion, and the cement paste adhesion could be better. Therefore, the addition of recycled aggregate will reduce the compressive strength. However, with the increase in the recycled aggregate replacement rate, the compressive strength of recycled concrete does not present a gradually decreasing trend [4,80]. The experimental results of Ma et al. [4] demonstrate that the compressive strength decreased to a maximum of 30.5% when the substitution proportion was 45%. The compressive strength decreased by 26.1% when the replacement ratio of recycled aggregate was 100%. The decrease was minimal when the replacement ratio was 75%, being only 11.6%, because the addition of recycled aggregate changes the aggregate gradation of concrete [89].

While Ma et al.’s [4] experiment was affected by other conditions, it did not consider nonlinear polynomial fitting. As for the fitting results, generally speaking, with the increase in the aggregate replacement rate of the BBA of RAC, the corresponding recycled concrete cube compressive strength showed a decreasing trend. The reason for this is that the production of recycled aggregate causes micro-cracks in the aggregate, and the recycled aggregate itself has a low strength and high brittleness and is far weaker than natural aggregate. Therefore, it is more likely to be damaged in the compression process, reducing the concrete’s strength. In addition to the fact that the replacement rate of BBA affects the strength of recycled concrete, the water–cement ratio is also essential, as displayed in Figure 3b. When the replacement rate of BBA is 30%, different water–cement ratios can also lead to changes in the cubic compressive strength of concrete in the order from low to high water–cement ratios, according to the data displayed in the figure, which are from Zhu et al. [9], Zong et al. [8], Chen et al. [80], MA et al. [4], and Zhao et al. [90]. As can be seen from the contrast in the figure, when the water–cement ratio is 0.4, the strength of the recycled concrete is the highest. As a whole, the strength of recycled concrete decreases with the increase in the water–cement ratio. When the water–cement ratio is high, there are relatively few cement particles in the concrete mixture, the distance between the particles is considerable, and the colloid produced via hydration is not sufficient to fill the space between the particles. In addition, too much water evaporates, leaving more pores and reducing the strength of the concrete.

### 3.2. Splitting Tensile Strength

**Figure 4:**
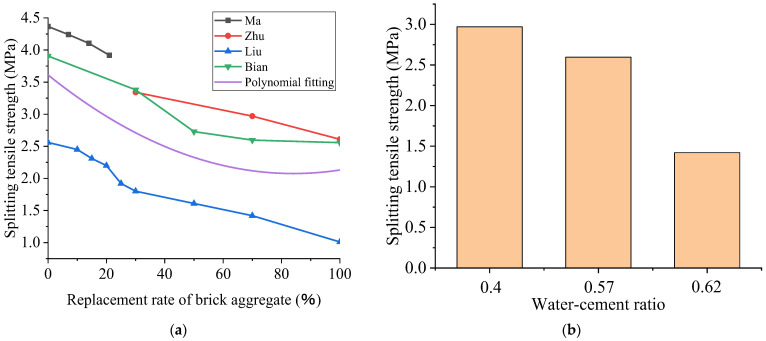
The effect of the recycled aggregate replacement ratio on the splitting tensile strength of recycled concrete (**a**) and the effect of the water–cement ratio on the splitting tensile strength of recycled concrete when the recycled aggregate replacement ratio is 70% (**b**) [5,7,9,91].

Figure 4a shows that the splitting tensile strength of recycled concrete decreases with the increase in the substitution rate of BBAs. For example, Bian et al. [91] conducted a comparison with a reference group with a 0% replacement rate. The splitting tensile strength of the RAC in the experimental groups with 30%, 50%, 70%, and 100% brick aggregate replacement rates are decreased by 13.6%, 30.4%, 33.8%, and 34.5%, respectively. When the substitution rate is 30%, the strength reduction is small, but when the substitution rate was more than 30%, the strength reduction is significant. The reason for this is that the strength of recycled aggregate of crushed brick is low, and there are many brick particles with low tensile strength and a larger number of surface cracks. The residual mortar on the brick particles’ surface leads to a weak bond strength, and most of the bricks are broken when the damage occurs. When the replacement rate of the broken brick is less than 30%, the skeleton of the concrete is coarse aggregate. When the replacement rate is less than 30%, the broken brick does not assume the primary skeleton role, and so the strength slowly decreases. When the replacement ratio is more than 30%, the broken brick begins to function as the skeleton of the matrix; thus, the strength of the recycled concrete begins to decrease significantly. Concerning the relevant results, the splitting tensile strength of recycled concrete decreases with the increase in the replacement ratio of BBA. When the replacement ratio of BBA is more than 30%, the decline in strength is more rapid. It tends to slow down when the substitution rate of BBA is over 70%, which corresponds to the results of Bian’s analysis [91].

The water–cement ratio also affects the splitting tensile strength of recycled concrete, as displayed in Figure 4b, which is derived from Zhu et al. [9], Bian et al. [91], and Liu et al. [5], in the order from low to high water–cement ratios. Under the condition of identical 70% replacement ratios of BBA, the splitting tensile strength is the highest when the water–cement ratio is 0.4. On the other hand, with the increase in the water–cement ratio, the splitting tensile strength gradually decreases. When the water–cement ratio is 0.62, the splitting tensile strength is merely 48% of that when the water–cement ratio is 0.4.

### 3.3. Flexural Strength

**Figure 5:**
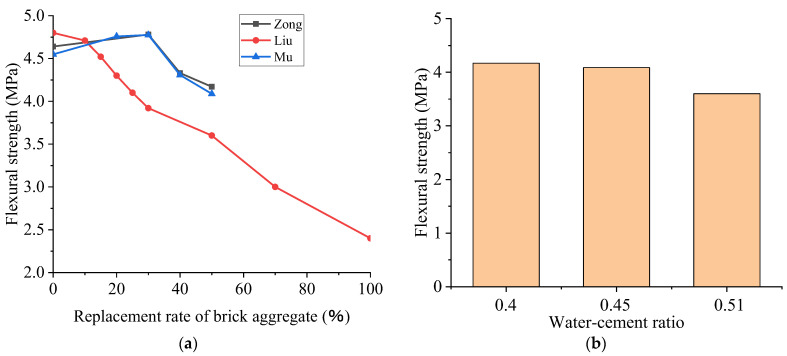
The effect of the recycled aggregate replacement ratio on the flexural strength of recycled concrete (**a**) and the effect of the water–cement ratio on the flexural strength of recycled concrete (**b**) when the recycled aggregate replacement ratio is 50% [5,8,92].

Figure 5a shows that the laws derived from different sets of experimental data are only partially consistent. The experimental data of Zonglan and Mu Chaohua demonstrate that the flexural strength of RAC first increases and then decreases with the increase in the BBA replacement rate. When the replacement rate of BBA is between 20% and 30%, the fracture strength of concrete is higher than that of the pure natural aggregate because the particle surface of BBA is relatively coarse. It has a better bond with the cement paste, which leads to a more robust bond surface. When the replacement ratio of BBA is between 40% and 50%, the flexural strength of RAC is less than 100%. The flexural strength of RAC is considerably diminished by the addition of BBA. The reasons for this are as follows:

- Because of the increase in the BBA, the internal strength of concrete is low;
- The quality of poor materials is increased, resulting in more easily damaged bond surfaces;
- It is indicated that the fracture resistance of recycled concrete will be diminished by the addition of too much BBA.

The results of polynomial fitting demonstrate that, as a whole, the flexural strength of RAC decreases with the increase in the replacement ratio of BBA. The higher the replacement ratio of BBA is, the faster the strength will decrease. Therefore, when the replacement rate of BBA is high, some measures should be taken to enhance the flexural strength of concrete.

As shown in Figure 5b, data were collected sequentially from Liu et al. [5], Zong et al. [8], and Mu et al. [92], illustrated in the order of low to high water–cement ratios with the identical BBA replacement rate of 50%. As can be observed from the contrast in the figure, when the water–cement ratio is 0.4, the flexural strength is the highest, and with the increase in the water–cement ratio, the flexural strength gradually decreases, which is consistent with the strength variation of natural aggregate concrete. On the other hand, the strength decreases with the increase in the water–cement ratio, but this decrease is slight. The strength of the concrete with a 0.51 water–cement ratio is 86.3% that of the concrete with a 0.4 water–cement ratio.

### 3.4. Modulus of Elasticity

**Figure 6:**
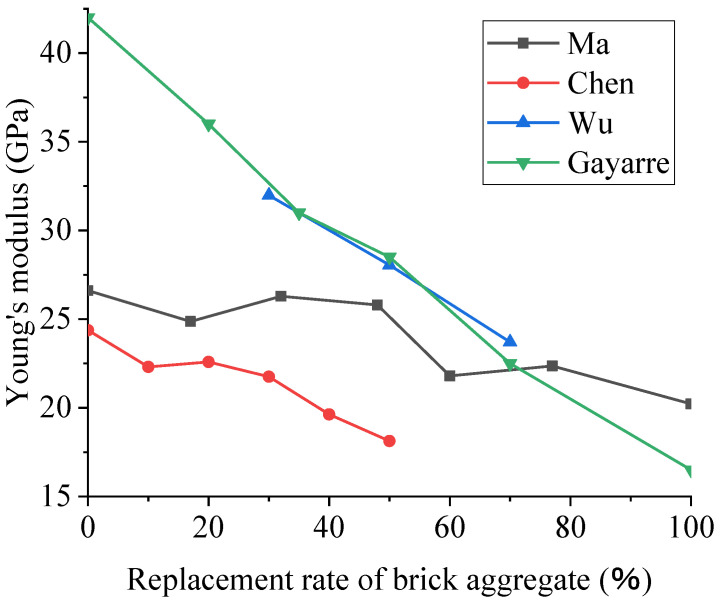
Effect of the brick aggregate substitution rate on the elastic modulus of RAC [4,63,75,80].

It is clear from the data in Figure 6 that the overall trend of the elastic modulus decreases with the increase in the replacement rate of brick aggregate. For example, in the experiment of Ma et al. [4], the elastic modulus of RAC with a 100% substitution rate was only 76.1% that of RAC without additional water and maintaining the same water–cement ratio. It can be observed that BBA has a higher crushing index and lower strength than NA. RAC has more defects and micro-cracks [3], and the attached old mortar has a higher porosity. When the specimen is compressed, the interface transition zone will produce a large deformation, which reduces the elastic modulus [4]. Therefore, the elastic modulus of recycled concrete will decrease with the increase in the substitution rate.

## 4. The Effect of Fiber Content on the Mechanical Properties of Recycled Brick Aggregate Concrete

It is essential to study the effects of different fiber contents on the mechanical properties of RAC. It is helpful to discover the law of the influence of the fiber content on the mechanical properties in order to determine a production plan that considers both economy and security in practical production.

### 4.1. Cubic Compressive Strength of Polypropylene-Fiber-Reinforced Recycled Concrete (28D)

**Figure 7:**
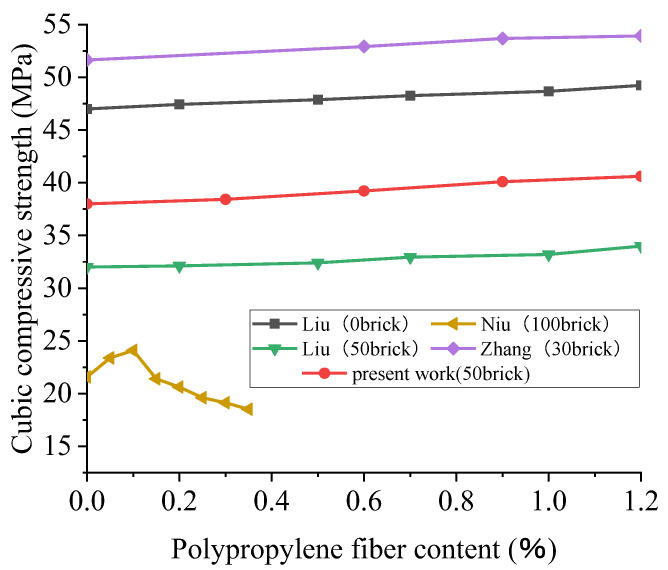
The change trend of the 28D cubic compressive strength of recycled concrete with the content of polypropylene fiber based on different contents of broken brick and the results of polynomial fitting [61,66,93].

Due to the need for reference data, the authors obtained experimental data. The designed water–cement ratio was 0.4, and the replacement rate of brick and aggregate was 50%. The volume fractions of polypropylene fiber were 0%, 0.3%, 0.6%, 0.9%, and 1.2%, respectively. A 28D cubic compression test was carried out on a 150 mm × 150 mm × 150 mm cubic specimen. It can be seen from the data comparison in Figure 7 that the cubic compressive strength of recycled concrete increased with the increase in the polypropylene fiber (PPF) content. When the replacement rate of BBA was greater than or equal to 50%, the RAC intensity decreased with the increase in the replacement rate of BBA. For example, in Liu’s experiment, the compressive strength of a 28D cube with 50% BBA was reduced by 32% when the fiber content was 0% compared to 0% BBA. This phenomenon is due to the low strength and bond strength of BBA. Thus, with the increase in the BBA content, the strength of recycled concrete decreases to a certain extent.

**Figure 8:**
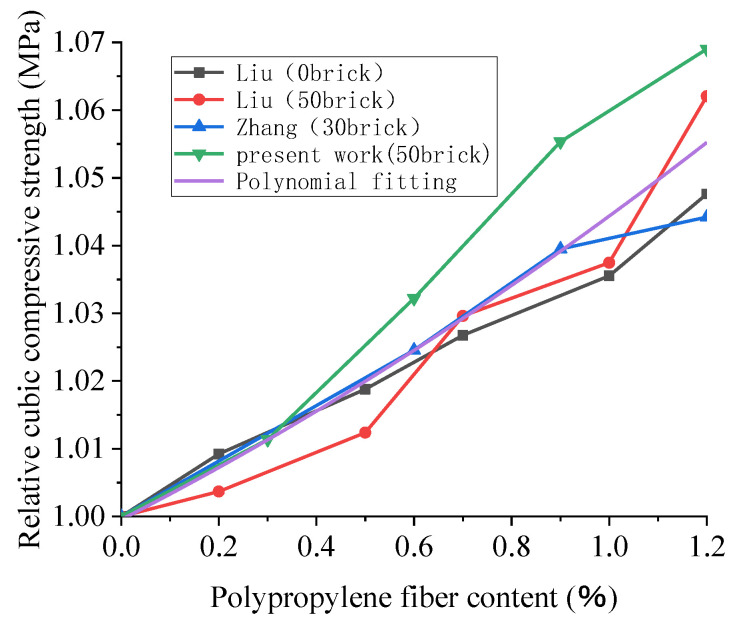
Effect of polypropylene fiber content on the 28D relative cubic compressive strength of recycled concrete [66,93].

As shown in Figure 8, the compressive strength of the recycled concrete cube is slightly increased by the polypropylene fiber when the content of broken brick is less than 50%, but the effect is insignificant. Additionally, taking Liu’s trial, when the content of broken brick is 50%, the contents of polypropylene are 0.2%, 0.5%, 0.7%, 1.0%, and 1.2%, and the compressive strength of the 28D cube is increased by 0.4%, 1.2%, 3.0%, 3.7%, and 6.3%, respectively. This is due to the addition of polypropylene fiber, filling the space between the aggregates so that the concrete is dense. Therefore, the recycled concrete’s cubic compressive strength is somewhat enhanced. However, the compressive strength of the recycled concrete is not significantly improved because of the low tensile strength of polypropylene fiber. Considering that Liu’s trial was affected by other conditions, this may be because of an error in the fiber content, the source of the recycled coarse aggregate, or the agitation method. For example, fibers’ capacity to enhance concrete performance under different loads depends on the type, size, aspect ratio, surface texture, and tensile strength of the fibers [94]. Unfortunately, these data are not provided. It can also be seen from Figure 7 that the fitting result of polynomial nonlinearity is good. The R^2^ of the fitting curve is 0.88. The cubic compressive strength of the recycled concrete increases with the increase in the substitution rate of polypropylene fiber, but the overall growth rate is slow. For recycled concrete’s cubic compressive strength, the improvement effect of polypropylene fiber is insignificant.

### 4.2. The Splitting Tensile Strength of Polypropylene-Fiber-Reinforced Recycled Concrete (28D)

**Figure 9:**
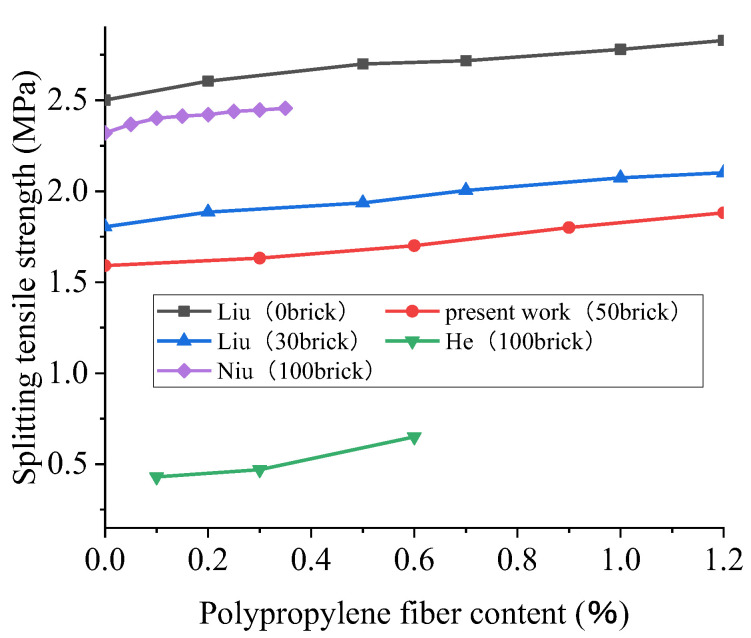
The effect of polypropylene fiber content on the 28D splitting tensile strength of recycled concrete with different brick contents and the results of polynomial fitting [61,93,95].

In order to enrich the experimental data and enhance the reliability of the analysis results, the authors obtained a set of experimental data. The trial applied a water–cement ratio of 0.4 and a replacement rate of brick-aggregate of 50%. The 28-day splitting tensile strength test was conducted on a cube specimen with glass fiber contents of 0%, 0.3%, 0.6%, 0.9%, and 1.2% and a size of 150 mm × 150 mm × 150 mm. As can be seen from the data in Figure 9, the addition of crushed brick decreased the splitting tensile strength of the recycled concrete, and the greater the addition was, the more noticeable the reduction in strength was. This finding agrees with the conclusion obtained from studies of the effect of the BBA replacement ratio on recycled concrete’s splitting tensile strength. Liu’s trial, for instance, demonstrated that when the fiber content was 0%, the splitting tensile strength of crushed brick was reduced by 30% compared with the control group (0%).

**Figure 10:**
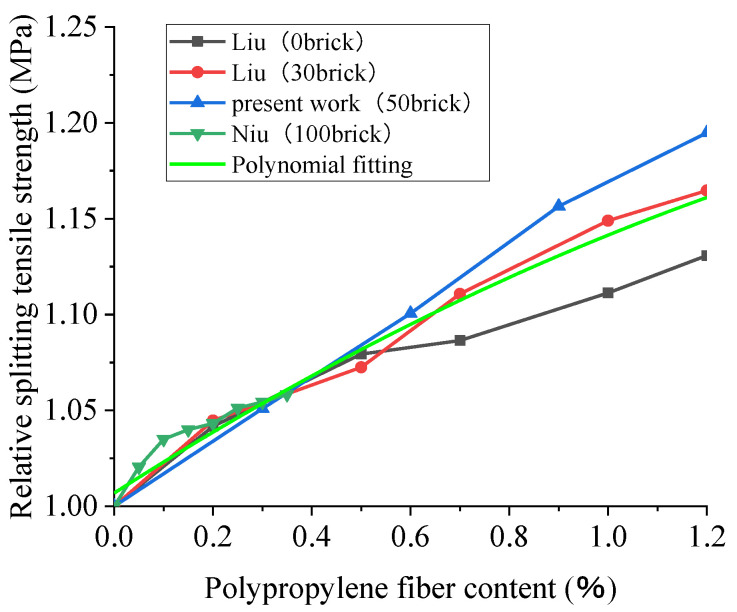
Effect of polypropylene fiber content on the 28D relative splitting tensile strength of recycled concrete [61,93].

Figure 10 shows that the trend of the influence of polypropylene fiber on the splitting tensile strength is the same with different contents of broken brick; the increase in the splitting tensile strength of polypropylene fiber is more significant than that in the compressive strength, and the higher content of broken brick is, the more pronounced the improvement is. For example, the splitting tensile strength increases by 3.9% and 11.4%, respectively, when the contents of broken brick are 0% and 30% and the volume fraction of polypropylene is 1.2%. The improvement of these properties can be accredited to the excellent tensile properties of the polypropylene fiber, the filling effect of the polypropylene fiber on the concrete, and its ability to endure specific tensile stresses and restrain the formation and development of cracks to a certain extent. Therefore, the splitting tensile strength of concrete is improved. Moreover, the fitting effect of the relative strength curve is better; the R^2^ is 0.93. From the relevant results, we can see thar the splitting tensile strength of the recycled concrete rises with the increase in the polypropylene fiber content. When the fiber content is lower, the splitting tensile strength of the recycled concrete increases with the increase in the polypropylene fiber content, the fitting curve is steep, and the splitting tensile strength increases rapidly. However, the slope of the curve decreases when the fiber content is more than 0.4%, and the rate of increase in the splitting tensile strength decreases with the increase in the fiber content.

### 4.3. Cubic Compressive Strength of Glass-Fiber-Reinforced Recycled Concrete (28D)

**Figure 11:**
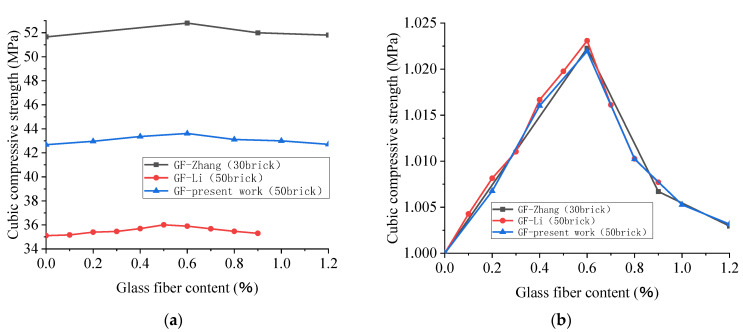
Effect of glass fiber content on 28D cubic compressive strength of recycled concrete with different contents of broken brick (**a**) and the effect of glass fiber content on the 28D relative cubic compressive strength of recycled concrete (**b**) [66,96].

Due to the lack of reference data, the authors obtained a set of experimental data, with the actual water–cement ratio of 0.4, BBA replacement rate of 50%, and size of 150 mm × 150 mm × 150 mm. Cubic specimens with glass fiber volume fractions of 0%, 0.2%, 0.4%, 0.6%, 0.8%, 1.0%, and 1.2% were subjected to cubic compression tests for 28 days. It can be seen from Figure 11a that the 28D cubic compressive strength of recycled concrete does not simply increase or decrease with the increase in the glass fiber content, and the strength shows little change under the influence of different broken brick replacement rates. It is evident from Figure 11b that the overall strength change tends to first increase and then decrease. When the fiber content is low, the increase in the fiber content performs a positive role of “Fine reinforcement,” raising the compressive strength. When the content of glass fiber is approximately 0.6%, the strength of recycled concrete reaches the maximum. When the content of glass fiber is more than 0.6%, due to the constraints of existing construction technology, the fiber in the concrete is bound to exist in the phenomenon of agglomeration. Due to the uneven distribution, resulting in difficulty in blending, the brick aggregate concrete’s internal pores increase. The more glass fiber there is, the more serious this phenomenon will be, and as a result, the strength of the recycled concrete gradually decreases. In addition, with the increase in the fiber content, the density of the structure will decrease, resulting in a decrease in the compressive strength of the cube. Furthermore, after the second crushing of the BBA, there are numerous internal cracks, and the initial defects are relatively severe. Many glass fibers weaken the capacity for bonding between the aggregates and magnify the original defects of the aggregate brick concrete. The fiber exceeds its role as a “Micro-reinforcing bar” in concrete, which leads to the weak bearing capacity of brick-aggregate concrete specimens with more pores under increasing external loads. Initial cracks appear and continue to extend along, and ultimately destroy, the pores.

**Figure 12:**
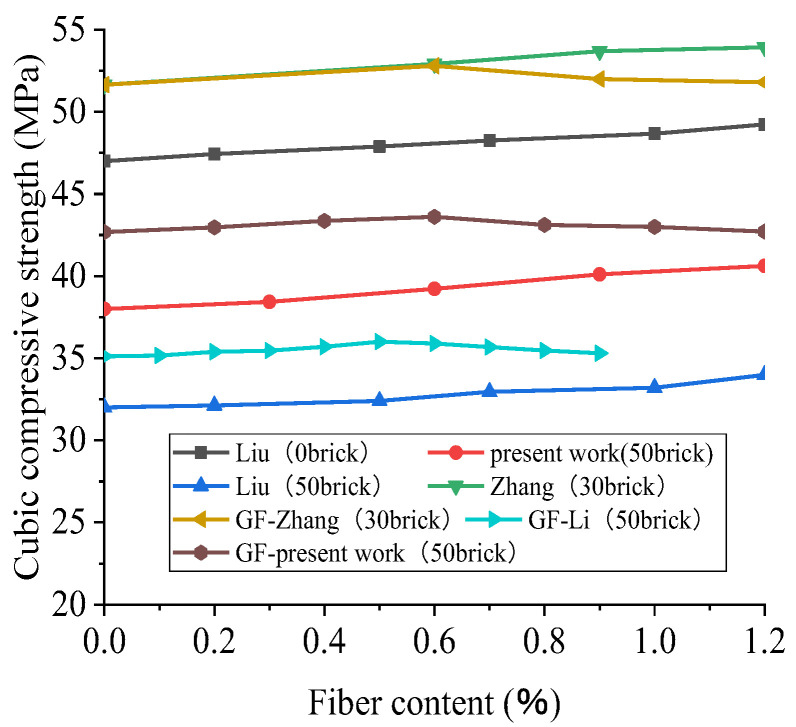
Cross-sectional comparison of the effects of fiber type and fiber content on the cubic compressive strength of recycled crushed brick concrete with different brick contents: GF [66,96]; PPF [61,66,93].

In Figure 12, the brown, yellow, and cyan data are the fiberglass data, and the remainder are the polypropylene fiber data. It can be seen from Figure 12 that the cubic compressive strength of recycled concrete rises with the increase in the fiber content when the content of broken brick is lower than 50%, but in the case of both GF and PPF, the improvement is not significant. When the content of broken brick is more than or equal to 50%, the cubic compressive strength of recycled concrete tends to rise at first and then decrease with the increase in the fiber content, according to the experimental data of Zhang [66], the present work, Li [96], and other researchers, showing that the cubic compressive strength of recycled concrete reaches its maximum when the fiber content is approximately 0.6%.

### 4.4. The Splitting Tensile Strength of Glass-Fiber-Reinforced Recycled Concrete (28D)

**Figure 13:**
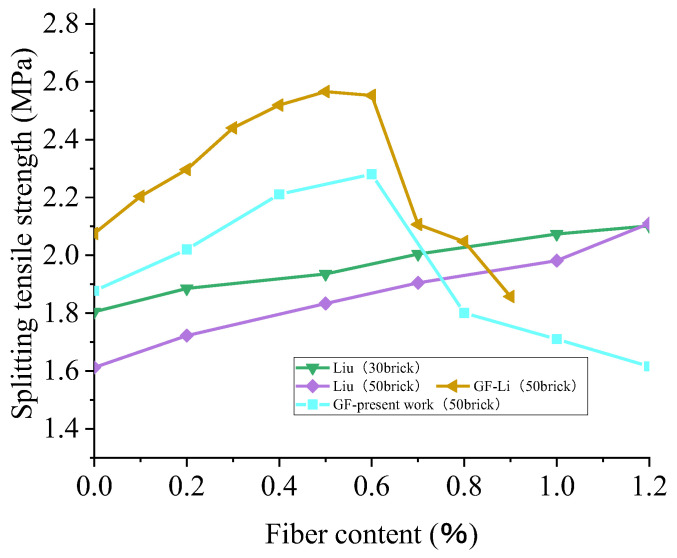
Transverse comparison of the effects of the fiber type and fiber content on the splitting tensile strength of recycled concrete with different contents of broken brick: GF [96]; PPF [61,93].

Due to the lack of reference data, the authors obtained a set of experimental data. The actual water–cement ratio was 0.4, the BBA replacement rate was 50%, and the volume fractions of glass fiber were 0%, 0.2%, 0.4%, 0.6%, 0.8%, 1.0%, and 1.2%. The cube specimen, with a size of 150 mm × 150 mm × 150 mm, was subjected to a crack tensile test for 28 days. The data in Figure 13 shows that with the increase in the glass fiber content, the splitting tensile strength of RAC first increases and then decreases, which is different from the effect of polypropylene fiber on the strength of recycled concrete. This may be because glass fibers are the equivalent of “Micro-reinforcement” in concrete. On the one hand, glass fibers can prevent or reduce the further growth of micro-cracks. On the other hand, the glass fibers can share the load with the concrete when the concrete reaches the peak load and breaks, and the fibers can still share part of the tensile force until it is eliminated. Therefore, the splitting tensile strength of aggregate brick concrete can be increased. If the glass fiber is added in proportions that are too high, the internal porosity of the concrete will be too high, and the density of concrete will not be guaranteed, but the splitting tensile strength will be reduced. The effects of different fiber contents on the splitting tensile strength are identical when the fiber content is lower, and the increase in the splitting tensile strength is more significant when the fiber content is 0–0.6%. In addition, the higher the content of broken brick is, the more prominent the reinforcement effect of the fiber is. Taking the data of Liu et al. [93] as an example, when the fiber content is 0.6%, the strength increases by 23.62% compared with that when the fiber content is 0%. However, when the fiber content is more than 0.6%, the strength of the broken brick increases by 23.62% compared with that when the content is less than 0.6%. With the increase in glass fiber content, recycled concrete’s strength declined. In contrast, the strength of polypropylene-fiber-modified recycled concrete shows the same growth trend. It can be inferred that the optimal content of glass fiber is less than that of polypropylene fiber.

## 5. Conclusions and Prospects

### 5.1. Conclusions

This paper reviewed the research status of glass-fiber- and polypropylene-fiber-reinforced recycled RAC. The effects of fiber on the mechanical properties of RAC were determined by considering factors such as the replacement rate of BBA, fiber type, and fiber content. Based on the literature review, the following conclusions can be drawn:

(1) Compared with natural coarse aggregate (NA), broken red brick aggregate (BBA) made of waste broken brick possesses a lower apparent density and bulk density, with a 19–40% lower apparent density and 20–47% lower bulk density, as well as a higher water absorption and crushing index, with an 11 times to 49 times higher water absorption and 2.2 times to 4 times higher crushing index, which directly indicates that broken red brick coarse aggregate is inferior in strength and stability to natural aggregate.

(2) For the optimal replacement rate of recycled concrete BBA, replacing natural aggregate with BBA has little effect on the compressive strength of the opposite side. The overall trend is downward, but it can fulfill the strength requirements of general engineering. Regarding the splitting tensile strength, when the replacement rate of BBA is less than 50%, the strength does not decrease significantly. However, when the replacement rate is more than 50%, the strength decreases considerably, which greatly influences the concrete. Regarding flexural strength, when the replacement rate of BBA is below 30%, it demonstrates a strengthening effect to a certain degree, which is worthy of attention. However, when the replacement rate is above 30%, the concrete strength shows a downward trend. In addition, with an identical aggregate substitution rate of the brick, the water–cement ratio also considerably affects the strength of concrete. When the water–cement ratio is more significant than 0.4, the compressive strength, splitting tensile strength, and flexural strength of the concrete decrease with the increase in the water–cement ratio. In addition, the elastic modulus of RAC decreases significantly with the increase in the replacement rate of brick aggregate, which indirectly indicates that the compressive strength of RAC is lower than that of ordinary concrete under the same water–cement ratio and other conditions. Therefore, on the whole, the mechanical strength of recycled concrete is less than that of ordinary concrete in all aspects when the replacement rate of BBA is higher. In addition, due to the defects of BBA, such as its large porosity, high water absorption, low strength, and bond strength, BBA significantly impacts recycled concrete’s mechanical properties and durability. In order to expand the application scope of recycled concrete in practical engineering, in addition to regulating the replacement rate of BBA and the water–cement ratio, the most useful method is to use fiber materials to produce fiber-reclaimed RAC with better mechanical properties.

(3) When the broken brick’s replacement rate is less than 50%, the cubic compressive strength and splitting tensile strength of the recycled concrete are enhanced by PPF. However, it should be noted that when the replacement rate of broken brick is higher, such as 100%, and the content of PPF reaches 0.2–0.3%, the strength of the recycled concrete shows a decreasing trend and starts to become lower than that of recycled concrete without PPF. Therefore, in practical application, it is essential to consider the comprehensive effects of the replacement rate of BBA and the fiber content.

(4) Glass fiber with high strength and good toughness has a better modifying effect on the recycled concrete of BBA: the replacement rate of BBA and the content of fiber have a compound effect on the strength of recycled concrete when the replacement rate of BBA is below 50%, and the cubic compressive strength of recycled concrete can be enhanced by adding an appropriate amount of GF, but this is not apparent. On the contrary, when the replacement rate of BBA is high and the replacement rate is 50%, the strength of recycled concrete will be reduced because of the addition of too much GF, as a large amount of alkali-resistant glass fiber weakens the ability for bonding between aggregates and magnifies the original defects of aggregate brick concrete, exceeding the strengthening effect of GF fiber on the cubic compressive strength. On the other hand, with a low replacement rate of BBA, when the content of GF is in a specific range (0–0.6%), the splitting tensile strength of recycled concrete can be enhanced.

(5) According to the present research, single-fiber-modified recycled RAC still needs to be improved, and this cannot ultimately improve the performance of RAC. Additionally, because of their high price, some high-performance fibers cannot be extended to the actual construction field. However, research on the hybrid-fiber-reinforced RAC, which can be produced through the positive hybrid effect of fiber, is still in the initial stage at home and abroad. Concrete’s fundamental mechanical properties, failure mechanism, and fiber-reinforced mechanism are still yet to be studied.

### 5.2. Prospects

Currently, in construction, recycled concrete can be utilized for roadsides, gutters [97], pavements [98], and island projects [99]. The recycled concrete of BBA is typically used in non-load-bearing members because of its characteristics. In recent years, the research and application of recycled aggregate concrete in beam columns, slabs, frame structures, and other load-bearing components have developed. Much research has been conducted on the flexural, static, and seismic behaviors of columns with large and small eccentricities and their mechanical behaviors, and researchers have studied the seismic performance of RBA and RAC panels [100]. The feasibility of the industrial application of recycled concrete has been evaluated in other studies, and the application of the assessment results in policy, technology, and marketing is feasible [101]. In addition, glass fiber has a stable market in the construction field worldwide [102]. Across the world, countries also attach great importance to the applications of polypropylene fiber [103]. In the future, with further research on hybrid fiber recycled concrete, progress in technology will provide broader application prospects for recycled concrete. At the same time, with the development of society, national governments’ laws and regulations, facilities, and technologies will be perfected, in addition to preferential policies and innovative management modes, and the public’s environmental awareness will gradually be enhanced. Enterprises in the field of environmental protection continue to innovate construction waste recycling technology, and construction waste recycling is becoming a prosperous industry. In this respect, the United States, Japan, Germany, and other countries have performed well [104]. Therefore, with further research and development in the construction waste industry, fiber-modified regeneration RAC will gain broad development prospects.

## Figures and Tables

**Table 1:** Basic performance index of broken brick and natural aggregate [78,79,80,81].

Type of Coarse Aggregate	Particle Size (mm)	Volume Density (kg/m^3^)	Apparent Density (kg/m^3^)	Water Absorption (%)	Crushing Index (%)
Natural aggregate (NA)	4.75~25	1432~1550	2615~2780	0.44~1.2	7.2~7.8
Broken red brick aggregate (BBA)	4.75~25	814~1149	1652~2130	13.24~21.7	17.14~28.5

## Data Availability

All data generated or analyzed during this study are included in this published article.
